# Effects of Residue Management on Decomposition in Irrigated Rice Fields Are Not Related to Changes in the Decomposer Community

**DOI:** 10.1371/journal.pone.0134402

**Published:** 2015-07-30

**Authors:** Anja Schmidt, Katharina John, Gertrudo Arida, Harald Auge, Roland Brandl, Finbarr G. Horgan, Stefan Hotes, Leonardo Marquez, Nico Radermacher, Josef Settele, Volkmar Wolters, Martin Schädler

**Affiliations:** 1 Department of Community Ecology, Helmholtz-Centre for Environmental Research—UFZ, Halle/Saale, Germany; 2 Department of Animal Ecology, Justus-Liebig-University, Giessen, Germany; 3 Crop Protection Division, Philippine Rice Research Institute, Muñoz, Nueva Ecija, Philippines; 4 iDiv—German Centre for Integrative Biodiversity Research Halle-Jena-Leipzig, Leipzig, Germany; 5 Department of Ecology, Faculty of Biology, Philipps-University, Marburg, Germany; 6 International Rice Research Institute, Los Baños, Metro Manila, Philippines; 7 J.F. Blumenbach Institute of Zoology and Anthropology, Georg-August-University, Goettingen, Germany; Chinese Academy of Sciences, CHINA

## Abstract

Decomposers provide an essential ecosystem service that contributes to sustainable production in rice ecosystems by driving the release of nutrients from organic crop residues. During a single rice crop cycle we examined the effects of four different crop residue management practices (rice straw or ash of burned straw scattered on the soil surface or incorporated into the soil) on rice straw decomposition and on the abundance of aquatic and soil-dwelling invertebrates. Mass loss of rice straw in litterbags of two different mesh sizes that either prevented or allowed access of meso- and macro-invertebrates was used as a proxy for decomposition rates. Invertebrates significantly increased total loss of litter mass by up to 30%. Initially, the contribution of invertebrates to decomposition was significantly smaller in plots with rice straw scattered on the soil surface; however, this effect disappeared later in the season. We found no significant responses in microbial decomposition rates to management practices. The abundance of aquatic fauna was higher in fields with rice straw amendment, whereas the abundance of soil fauna fluctuated considerably. There was a clear separation between the overall invertebrate community structure in response to the ash and straw treatments. However, we found no correlation between litter mass loss and abundances of various lineages of invertebrates. Our results indicate that invertebrates can contribute to soil fertility in irrigated paddy fields by decomposing rice straw, and that their abundance as well as efficiency in decomposition may be promoted by crop residue management practices.

## Introduction

Establishing sustainable agricultural practices together with the restoration of functional food webs for integrated pest management and nutrient cycling has become a major focus of current rice research. Decomposition in general, and decomposition of rice straw in particular, is an important process regulating energy flows and nutrient cycles in rice paddies [1–3]. Therefore, the establishment of a functional decomposer community is essential in the development of practices for sustainable agricultural management in rice dominated landscapes [4]. However, to date, decomposition dynamics (e.g. interactions between microbes and invertebrates) as well as detritivore community assembly in flooded rice ecosystems have received little research attention. The present study examines the effects of different crop residue management practices on invertebrate communities and decomposition rate in tropical rice fields.

Rice straw decomposition by invertebrate decomposers is likely to be of particular importance to the stable and adequate availability of nutrients and to sustained soil quality in rice fields, because under the anaerobic conditions created by flooding microbial decomposition rates are expected to be low [5–10]. Therefore, major component of our study was to evaluate the contribution of invertebrates to litter decomposition in tropical irrigated rice fields. However, the complex relationship between soil biodiversity and ecosystem function is poorly understood [8, 11–14]. The effects of soil invertebrates on litter decomposition are often rather indirect; nevertheless, the activity of soil invertebrates is an essential determinant of decomposition rates and nutrient release [10, 15–17]. For example, litter fragmentation by invertebrates enhances microbial decomposition by increasing the surface area of plant fragments which creates a more stable and favorable micro-environment for decomposer microbes [1, 18].

The activity of invertebrate and microbial decomposers depends primarily on moisture and temperature [18–22], but also depends on the quality of the litter, e.g. lignin concentrations [23] and C/N ratios [3, 15]. Decomposers generally prefer high quality substrates with low C/N ratios, which results in faster decay rates [9, 24, 25]. Rice straw residues have high C/N ratios (approx. 61, see S1 Fig) compared to litter from other herbaceous plants (e.g. ranging from 19 to 30; see [26]); nevertheless, rice straw represents an important carbon and nitrogen source in rice paddies [20, 27]. Crop residues are often burned by farmers for cost-effectiveness or for the lack of alternative technology to incorporate large amounts of residues into the soil [18, 28, 29]. This results in a loss of both C and N [20]. Recently, an awareness of the importance of rice straw for nutrient supply has led to increasing efforts to improve strategies of crop residue management [18, 30]. Several studies have indicated that rice straw increases the content of mineralized N in the soil [31–33] and can improve crop yields (e.g. [34]). Therefore, the incorporation of rice straw residues into the paddy soil can further reduce N losses and enhance N availability for plants [35].

Rice fields provide habitat for a bewildering variety of soil-dwelling and freshwater animals [36, 37]. The most abundant groups of invertebrates involved in decomposition in flooded rice fields are oligochaetes, like *Enchytraeidae* or *Tubificidae*, chironomid larvae, nematodes and microcrustaceans. As an essential driver regulating nutrient cycling processes [1, 38] these organisms may increase paddy soil fertility [39]. In contrast, soil-dwelling microarthropods, like *Acari* or *Collembola*, occur in comparatively low numbers and are therefore thought to have limited influence on decomposition processes in irrigated rice [38, 40, 41].

Under aquatic conditions, litter initially decomposes at a fast rate due to leaching of water soluble substances [1]. It has been repeatedly demonstrated for flooded rice fields that after this initial rapid phase with large reductions in litter biomass, decomposition rates eventually slow down as soluble components become exhausted [14]. Deceleration of decomposition in tropical aquatic systems may also be due to a gradual decrease in fungal biomass over time [9], which makes the litter less attractive for decomposer organisms, especially grazers.

The results of previous studies that examined the effects of crop residue management on faunal diversity and abundance have been generally inconsistent. There is general agreement that management practices in flooded paddy fields do not affect the species richness of the aquatic and soil fauna [42, 43], but do affect relative abundance and, therefore, the composition of these species assemblages. Friebe and Henke [44] indicated that greater tillage intensity was associated with significantly lower abundances of soil fauna, which also decreased rates of straw decomposition. In contrast, Singh et al. [18] presented evidence of a positive effect of the incorporation of crop residues into soil on populations of all groups of macro- and microorganisms. Therefore, in the present study, we examine how management practices influence the composition and abundance of different functional groups of aquatic and soil fauna and whether this fauna contributes to straw decomposition in tropical flooded rice fields. For this, we investigated common practices of residue management that differed in the materials applied (ash *vs*. straw) and the mode of application (scattering on the surface *vs*. incorporating in the soil).

We tested the following hypotheses:

(1) Invertebrates contribute significantly to the mass loss of rice straw in paddy fields, with (2) the abundances of invertebrates being higher in rice fields with straw amendment compared to ash treatment. (3) This effect depends on the mode of residue application since scattering residues on the surface might favor different groups of invertebrates (henceforth lineages) from those favored when the material is incorporated into the soil. (4) Abundances of functional groups of invertebrates and their relative contribution to decomposition will vary over time with stronger effects of invertebrates and faster decomposition rates at the beginning of the season.

## Materials and Methods

### Study site

As part of the LEGATO project [45] our decomposition experiment was conducted at the field research station of the Philippine Rice Research Institute (PhilRice) in Muñoz, Nueva Ecija province on the island of Luzon, Philippines (elevation: 50 m above sea level; latitude 15.67, longitude 120.89 WGS84 decimal degrees; LEGATO region: PH_2, [46]). The soil in this area is of volcanic origin with a high proportion of clay and loam. In this region, lowland flooded rice is mostly cultivated in two crop cycles per year, one in the dry season (January—April) and one in the wet season (June—September). Our study was carried out during the dry season of 2013. During the experiments, the average temperature was between 25.8°C and 29.6°C, and the monthly rainfall varied between 0 and 2 mm (weather data provided by PhilRice—Central Experiment Station). The experimental site had previously been used for wet-rice cultivation for about 50 years. As the experiment was conducted at the field research station of the Institute, the rice fields of our study were not “true” farmers' fields, but were created just for this experiment. The experiment was carried out with the permission of and in cooperation with researchers of PhilRice. Our study did not involve any endangered or protected species.

### Study design

The experiment was arranged as a randomized complete block design with five blocks, each with five plots, and arranged in a 5 × 5 grid (S2 Fig). Each plot had a surface area of 25 m^2^. Four crop residue management treatments were applied randomly to the plots within each of the five blocks four days prior to planting the rice seedlings. Treatments included: ash of burned rice straw scattered on the field (henceforth abbreviated by ‘Asc’), ash of burned rice straw mixed in the soil (‘Ami’), rice straw scattered on the field (‘Ssc’) and rice straw mixed in the soil (‘Smi’). In the control plots (‘Ctr’), no ash or straw was added. For each treatment 10 tons ha^-1^ of rice straw were either burned or pre-decomposed (scattered in the field for 8 weeks during the fallow period and then put into sacks at the green house where it was sprinkled with water once a day for another 3 weeks) to simulate field conditions before application to the experimental plots.

Rice seed (*Oryza sativa* L., variety NSIC Rc 222) was sown in dry seedbeds until the seedlings were 27 days old at which time they were transplanted to the plots. The rice crop was managed according to local farmers’ practices, including mechanical plowing of dry soil one month before transplanting the rice seedlings (after ca. 8 weeks fallow period), flooding and harrowing of the field two weeks before transplanting (from then on the field was kept flooded until harvesting), and leveling of the soil surface five days before transplanting. Molluscicides (*‘Bayluscide’*—active ingredient Niclosamide) and herbicides (*‘Machete’*—active ingredient Buthachlor) were applied shortly after transplanting. Fertilizer (*‘Swire’* 14-14-14 with urea 46-0-0) was applied two times (7 and 30 days after transplanting) and no insecticides were applied during the experiment. The rice plants were harvested 82 days after transplanting.

#### Litterbags

In order to quantify the contribution by invertebrate decomposers to total rates of decomposition we used nylon litterbags of 15 cm × 20 cm with two different mesh sizes [47] that were filled with 10 g of air-dried, chopped rice straw (*Oryza sativa* L., variety NSIC Rc 222) and fixed to the ground by coarse nylon nets and bamboo sticks. Subsamples of the straw were retained for initial moisture and chemical analyses. The litterbags were set in the field one day after transplanting the rice seedlings. The fine-meshed litterbags had a mesh size of 20 μm × 20 μm and allowed access of microbes and part of the microfauna (e.g., fungi, bacteria, protozoa, micro-nematodes.; henceforth referred to as ‘microbial decomposition’). The coarse-meshed bags had a mesh size of 5 mm × 5 mm and allowed access of most of the invertebrate groups [48]. The litter mass losses in our fine-meshed bags represented microbial driven decomposition, since microarthropods show comparatively low abundances in flooded rice agriculture [41] and therefore are assumed to have negligible influence on the decomposition process [38, 40]. Litterbags were retrieved after 25 days, 50 days and 75 days of exposure in the field. The two types of litterbags were arranged pair-wise on the soil surface with a maximum spacing of 2 cm between bags, with three replicates of one fine-meshed and one coarse-meshed litterbag per block per treatment (management practice) per retrieval time (total number of bags: 450). The litterbag pairs were randomly spread within the fields. After retrieval of the bags, soil particles, roots, and other alien plant material adhering to the straw were removed. The cleaned straw was dried at 60°C for at least three days and weighed to the nearest centigram to calculate litter mass losses. The C and N contents of the original straw as well as retrieved straw from each litterbag were determined using an Elementar Vario EL element analyser (Elementar Analysengeräte GmbH, Hanau, Germany).

#### Soil invertebrates—Sampling and level of identification

Soil invertebrates were sampled from all 25 plots. The field sampling was carried out at 25 and 75 days after the start of the litterbag experiment. On each date, five soil core subsamples (approx. Ø 2 cm, 10 cm depth) were taken per plot for the extraction of nematodes following a modified Cobb’s decanting and sieving method [49]. The nematodes were identified to genus level and assigned to feeding groups [50]. For the mesofauna, two soil cores (approx. Ø 5 cm, 10 cm depth) were taken. Microarthropods were extracted using a MacFadyen high-gradient extractor [51], and were sorted, counted and identified to suborder or family level. The second mesofauna soil core sample was manually sieved and decanted for the extraction of *Enchytraeidae* (potworms), which were suspended in 70% ethanol and counted. Additional cores (Ø 5 cm, 10 cm depth) were used for the analyses of abiotic soil parameters.

#### Aquatic invertebrates—Sampling and level of identification

Aquatic meso- and microfauna were taken from all 25 plots using dip nets (Ø 20 cm) of 0.8 mm mesh size. Sampling was carried out at the middle of the rice cycle (50 days after the start of the experiment) with a single sweep of 5 m length taken along the middle of each plot. The sampled invertebrates were directly transferred to 70% ethanol, sorted and identified to family or order level. We use the term “lineage”, which refers to phylogenetic groups differing in their taxonomic hierarchy in the remainder of the text, because soil and aquatic invertebrates were determined to different taxonomic levels.

### Data analyses

A general linear mixed model (GLMM) Type III sum of squares (procedure MIXED, SAS 9.2) was used to analyze litter-, C- and N mass loss as well as the relative contents of C and N (split-split-plot ANOVA) in relation to *‘treatment’* (crop residue management method; 5 levels within main plot), *‘time’* (retrieval time of litterbags; 3 levels within sub plot), and *‘mesh’* (mesh size of litterbags; 2 levels within sub-sub plot) as well as their interactions. The factors *‘block’* (5 levels within main plot) and *‘replicate’* (3 levels within sub plot) were considered random. Soil fauna data (split-plot ANOVA) were analyzed according to *‘treatment’* and *‘time’* (soil core sampling dates; 2 levels within sub plot), and also including *‘block’* as a random factor. The aquatic fauna (one-way ANOVA) was analyzed in a similar way, but excluding the factor *‘time’*. Post hoc Tukey’s HSD tests were carried out to reveal significant differences between the respective factor levels within factors.

For analyses of community structure, average values of the two sampling dates for lineages of soil invertebrates were calculated. For comparison between sampling methods (soil core *vs*. dip net), abundances of the lineages were standardized using z-transformation. To evaluate relations between abundances of the lineages and the management methods (*‘treatment’*) a redundancy analysis—RDA [52, 53] was carried out using R 2.1.4.2., package *vegan* [54]. We used this specific multivariate method, which requires linear relationships between lineages as well as between assemblages and environmental variables (Euclidean metric), because of the homogeneity in our community dataset and the short environmental gradient [55, 56]. According to Lepš and Šmilauer [57] the use of linear methods is appropriate, if the longest gradient, calculated using DCA/DCCA, is smaller than 3; in our dataset the longest gradient was 0.8.

Analyses of co-variance (ANCOVA) were used to analyze the relationships between litter mass losses and selected aquatic and soil-dwelling lineages including the same fixed and random factors as in the above described ANOVAs on litter mass losses. Lineages were included successively as covariates to reveal linear relationships of variances. Combining all independent and measured variables from our experiment, we used structural equation models (SEM) to test for direct and indirect interaction effects between observed endogenous variables (= independent variables) and exogenous predictor variables (= fixed factors).

## Results

### Rice straw decomposition

**Fig 1:**
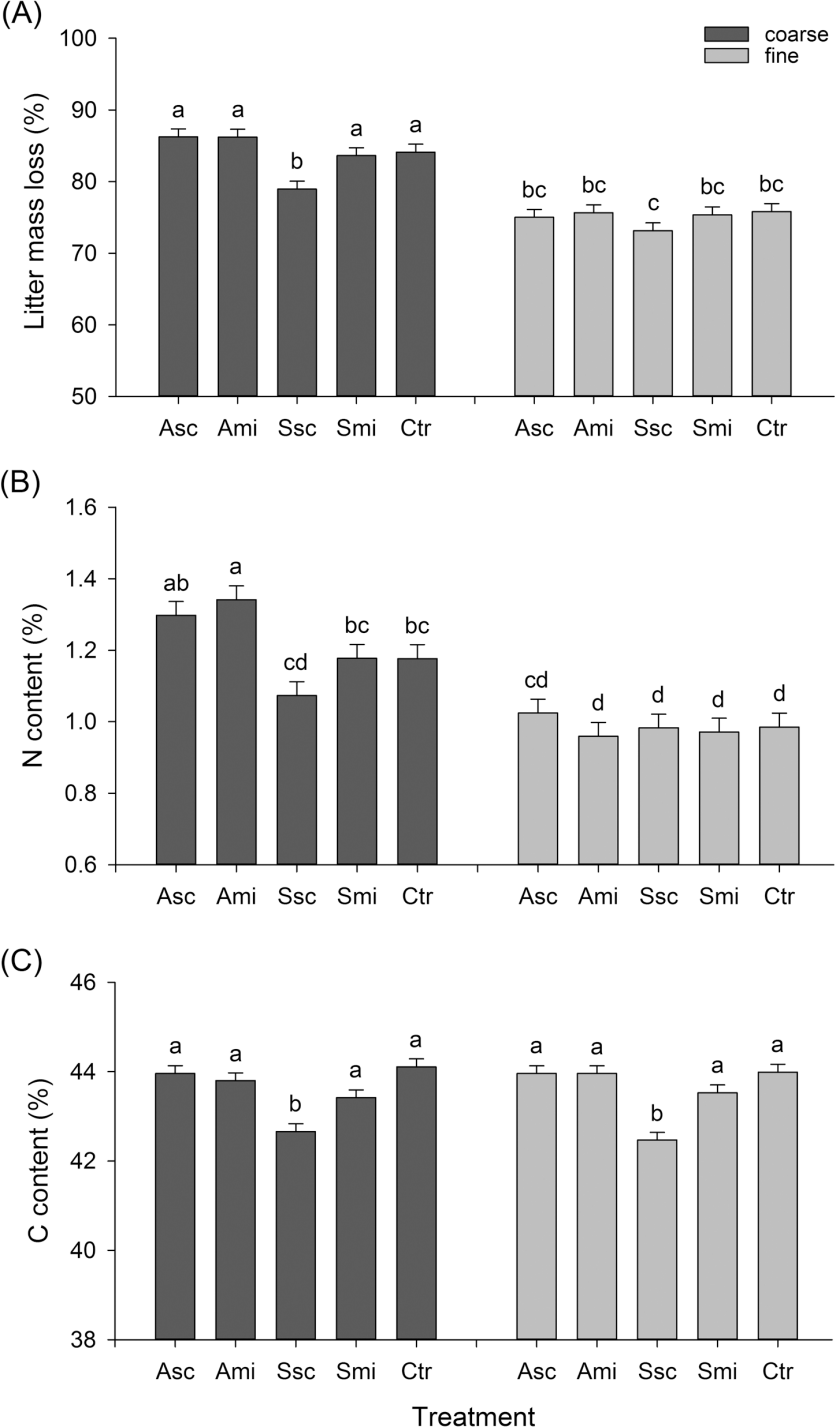
Litter mass loss, C content, N content: mesh size × treatment. Percent litter mass loss (A), N content (B) and C content (C) (means + standard error SE) of rice straw retrieved after the five treatments in coarse-meshed (decomposition by invertebrates and microorganisms) and fine-meshed (decomposition by microorganisms) litterbags. Different letters above the bars indicate significant differences between means (Tukey’s HSD, *P ≤ 0*.*05*). Values of the original straw (= *time 0d*): N = 0.6%, C = 36.8%. Treatment abbreviations: ‘Asc’—ash of burned rice straw scattered on the field, ‘Ami’—ash of burned rice straw mixed into the soil, ‘Ssc’—rice straw scattered on the field, ‘Smi’—rice straw mixed into the soil, ‘Ctr’—control (no ash or straw added).

**Fig 2:**
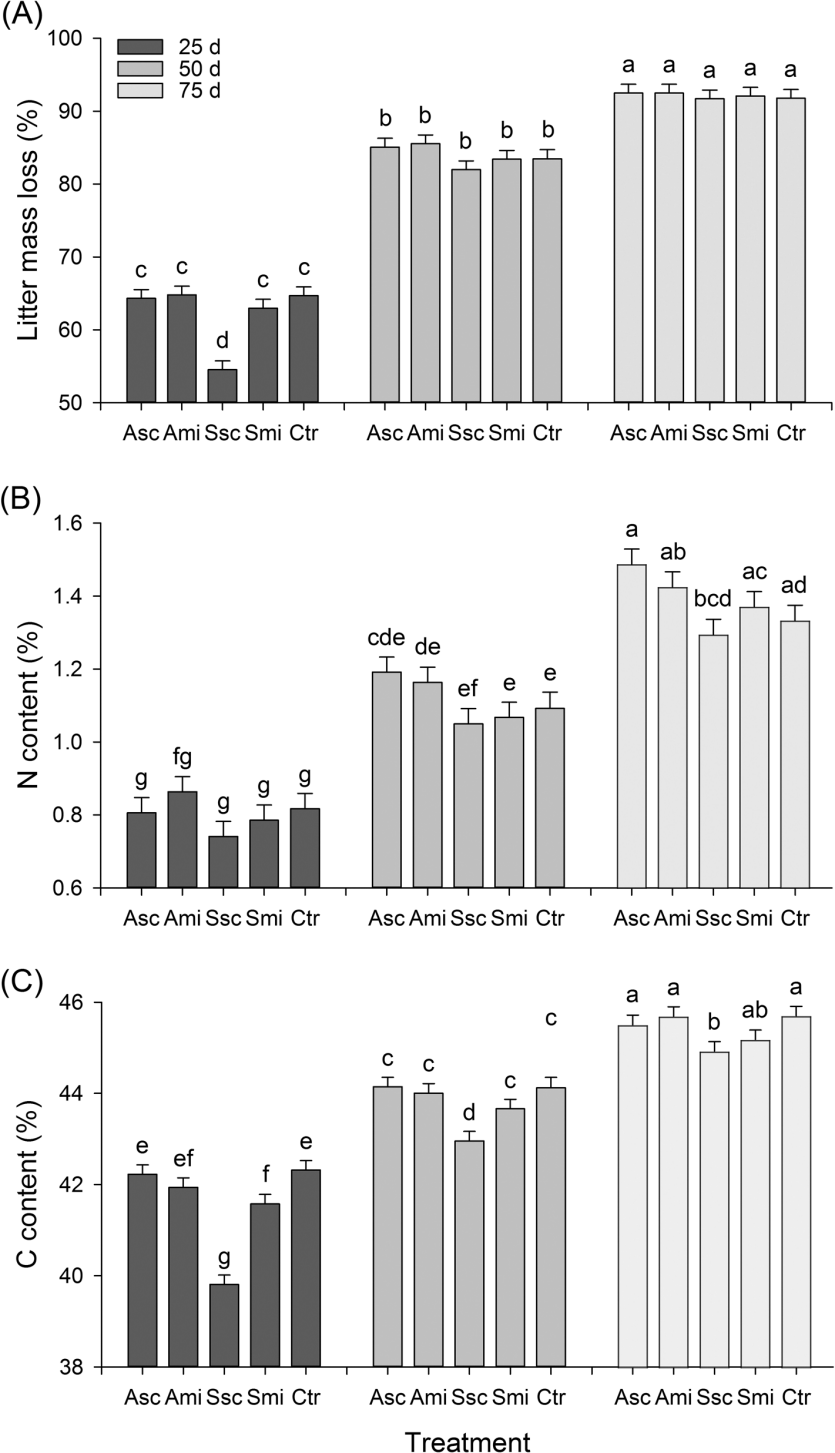
Litter mass loss, C content, N content: treatment × time. Percent litter mass loss (A), N content (B) and C content (C) (means + SE) of rice straw litter retrieved from the five treatments at three points in time. Different letters above the bars indicate significant differences between means (Tukey’s HSD, *P ≤ 0*.*05*). Values of the original straw (= *time 0d*): N = 0.6%, C = 36.8%. For abbreviations see Fig 1.

The mean loss of litter mass in coarse-meshed litterbags was higher than in the fine-meshed bags (84 ± 0.8% *vs*. 75 ± 0.8% (overall mean across all treatments and retrieval times ± SE) respectively; Table 1). All tested factors as well as their two-way interactions showed highly significant effects on litter mass loss (Table 1). Mesh size had a significant effect on litter mass loss on all retrieval dates (Table 1, S3A Fig). The mean percent litter mass loss was lower from the coarse-meshed bags (Fig 1A) retrieved after 25 days (Fig 2A) in plots with straw scattered on the surface of the field (treatment Ssc) compared to the other treatments. In both the fine- and coarse-meshed bags, retrieved later in the rice cycle, the mass loss of rice straw litter was similar across all five treatments (Fig 2A).

**Table 1:** The effects of *‘treatment’*, *‘time’* and *‘mesh’* and their interactions on litter mass loss of rice straw and the N and C contents of the retrieved straw using a GLMM type III sum of squares. Significant effects are indicated in bold font.

Factors	Litter mass loss (%)			N content (%)			C content (%)		
	Df	F	*P*	Df	F	*P*	Df	F	*P*
*treatment*	4,16	6.31	**0.003**	4,16	3.00	**0.05**	4,16	23.5	**< .0001**
*time*	2,187	2053	**< .0001**	2,187	579.72	**< .0001**	2,187	562	**< .0001**
*mesh*	1,206	652	**< .0001**	1,206	266.43	**< .0001**	1,206	0.01	0.91
*treatment × time*	8,187	8.47	**< .0001**	8,187	1.52	0.15	8,187	4.56	**< .0001**
*treatment × mesh*	4,206	7.78	**< .0001**	4,206	11.99	**< .0001**	4,206	0.65	0.63
*mesh × time*	2,206	61.5	**< .0001**	2,206	24.75	**< .0001**	2,206	36.2	**< .0001**
*treatment × mesh × time*	8,206	1.27	0.26	8,206	1.18	0.31	8,206	2.23	**0.03**

Factor *‘treatment’* represents the five different management practices (Asc, Ami, Ssc, Smi, Ctr; for abbreviations see Fig 1), the factor *‘time’* is the effect of the three different time periods for which the bags were left in the fields (25d, 50d, 75d), and factor *‘mesh’* the two mesh sizes (5 mm and 20 μm) used in every plot. The model also includes the random effects of the factors *‘block’* and *‘replicate’*; these two factors and their interactions are not shown.

Except for the *treatment × time* interaction, N content of the retrieved rice straw litter was also affected by all tested factors and their two-way interactions (Table 1). The same applies to C contents, except that there were no significant effects of *mesh* and the *treatment x mesh* interaction (Table 1). Similar to the total litter mass loss, N and C contents were lowest in the Ssc-plots (Fig 1B and 1C). Furthermore, the difference of C contents between Ssc and the other four treatments was largest for straw in litterbags retrieved after 25 days (Fig 2C). Whereas C and N contents of straw increased with time, C/N ratios decreased (Table A in S1 File, S1B Fig). This accounts for the relatively slower loss of N compared to C during decomposition.

There was a higher increase in N content over time in coarse-meshed compared to fine-meshed litterbags, but a higher increase in C content over time in fine-meshed bags (S3B Fig and S3C Fig). C/N ratios also followed this pattern, with higher values for straw in the fine-meshed compared to coarse-meshed bags (S1A Fig). When comparing the mass losses of C and N from rice straw litter retrieved from the five treatments, similar patterns emerged as for litter mass loss and relative C and N contents: firstly, there were no differences within fine-meshed bags; secondly, C and N mass loss were lower in coarse-meshed bags of treatment Ssc compared to the other four treatments; and thirdly, towards the end of the season differences between the treatments were negligible (Table B in S1 File, S4 Fig).

### Rice yield

**Fig 3:**
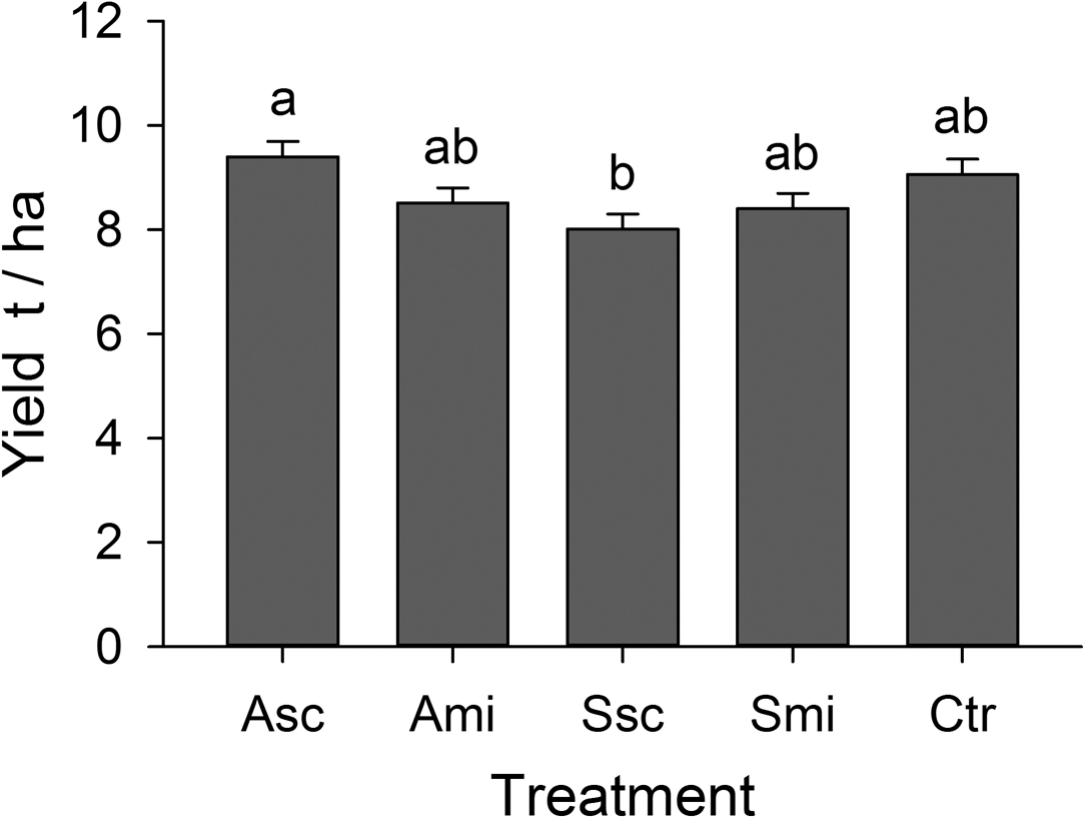
Rice yields. Yields per *‘treatment’* calculated from an adjusted grain weight at 14% moisture content (means + SE); different letters above the bars indicate significant differences between means (Tukey’s HSD, *P ≤ 0*.*05*). For abbreviations see Fig 1.

Rice yields were affected by crop residue management practices; minimum yields were obtained from fields where the rice straw was scattered on the soil surface, but this was not significantly different from the control plots (F_4,16_ = 3.6, *P = 0*.*29*; Fig 3).

### Soil and aquatic invertebrates

**Fig 4:**
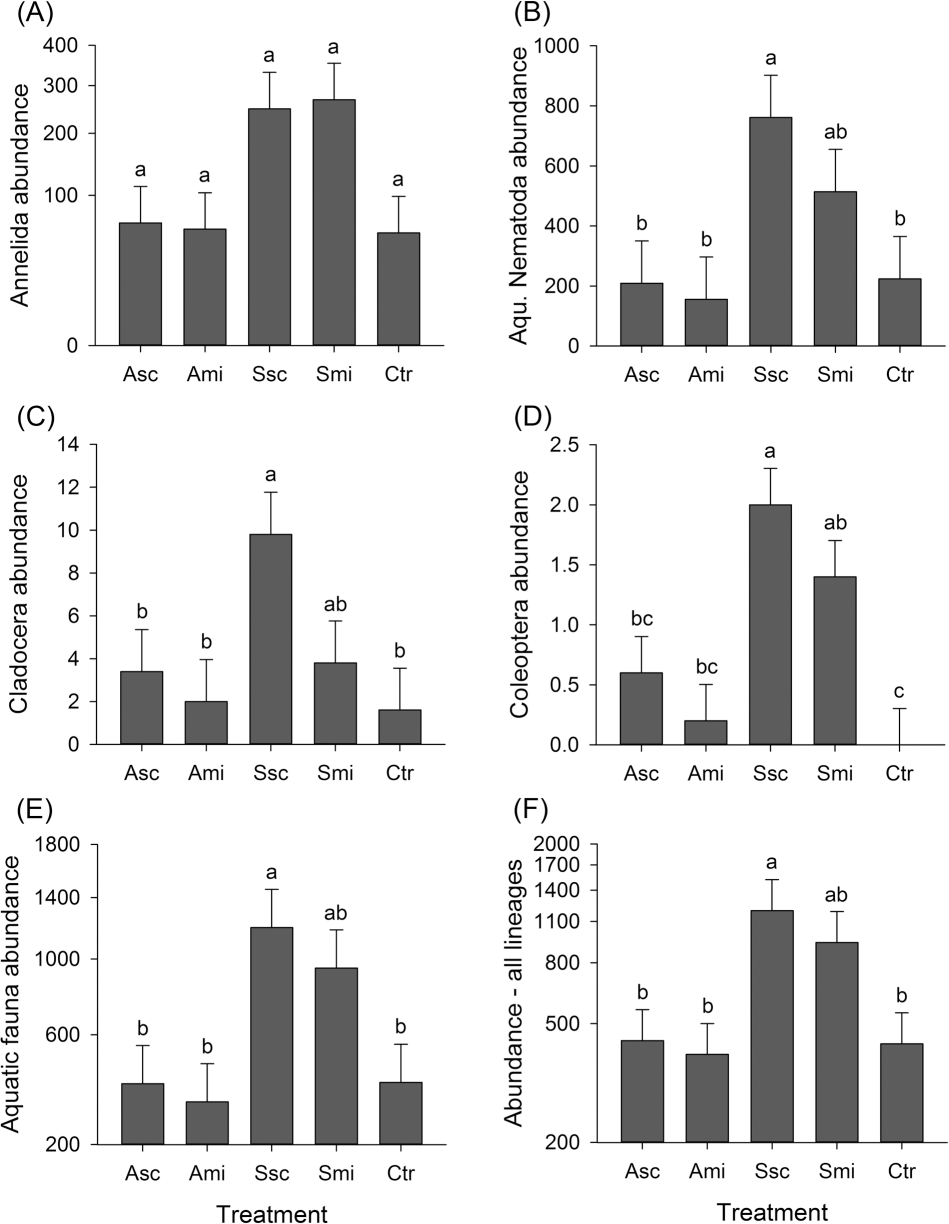
Abundance of aquatic fauna. Abundances of aquatic fauna groups and all lineages per *‘treatment’* (number of individuals). Panel (E) shows the sum of all aquatic fauna samples and panel (F) shows the total numbers of aquatic fauna together with soil fauna (means + SE); different letters above the bars indicate significant differences between means (Tukey’s HSD, *P ≤ 0*.*05*). For the number of annelids the post-hoc test revealed no significant differences in means. For abbreviations see Fig 1.

Crop residue treatment significantly affected the abundances of selected aquatic lineages as well as the sum of all lineages (aquatic + soil) (Table 2) with the highest abundances recorded at fields with straw amendment (treatments Ssc and Smi; Fig 4). There was no significant treatment effect on functional groups of soil invertebrates alone (Table C in S1 File). However, abundances were significantly different between the two sampling dates (S5C–S5G Fig). Most mesofaunal lineages, like the *Acari*, were more abundant at the beginning of the rice cycle. Fluctuations in nematode abundances varied between the feeding guilds: plant-feeding nematodes were most abundant in the beginning, but omnivorous nematodes were most abundant towards the end of the rice cycle. There was also a significant time effect on soil parameters (soil pH & soil organic C content, Table C in S1 File, S5A Fig and S5B Fig), but there were no significant treatment effects.

**Table 2:** The effect of *‘treatment’* on selected aquatic fauna groups and the sum of all lineages using a GLMM type III sum of squares. Error df = 16; significant effects are indicated in bold font.

Factor	Annelida abundance (sqrt)			Aquatic Nematoda abundance			Cladocera abundance		
	Df	F	*P*	Df	F	*P*	Df	F	*P*
*treatment*	4	3.48	**0.03**	4	4.12	**0.02**	4	4.68	**0.01**
	Coleoptera abundance			Aquatic fauna abundance (sqrt)			Abundance—all lineages (ln)		
	Df	F	*P*	Df	F	*P*	Df	F	*P*
*treatment*	4	7.70	**0.001**	4	4.64	**0.01**	4	5.09	**0.01**

Factor *‘treatment’* represents the five different management practices (Asc, Ami, Ssc, Smi, Ctr; for abbreviations see Fig 1). “Abundance—all lineages” refers to the total numbers of aquatic and soil invertebrates. The model also includes the random effect of the factor *‘block’*; which is not shown; ‘sqrt’—data square root transformed, ‘ln’—data log_e_ transformed.

**Fig 5:**
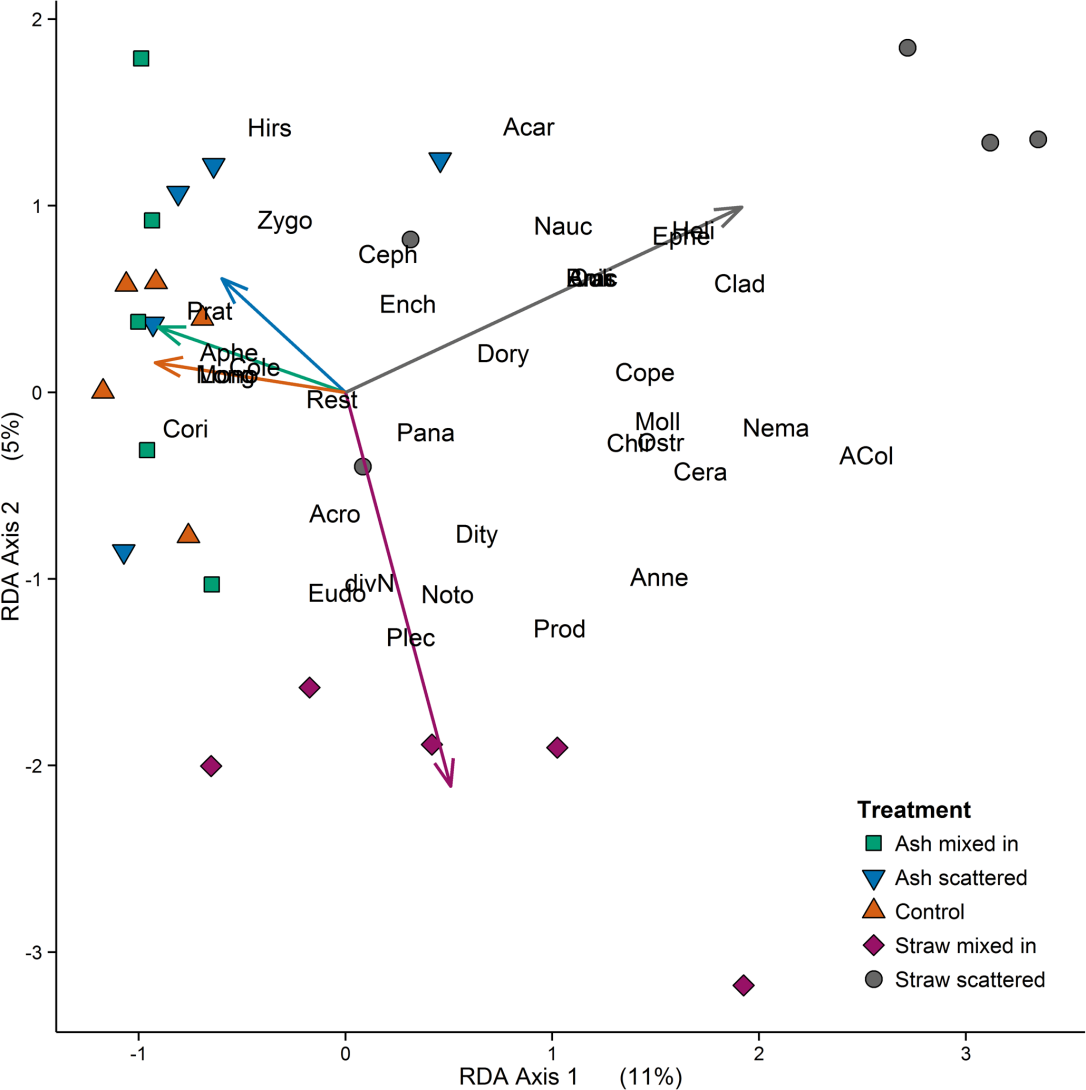
RDA plot including all lineages. Euclidean distance biplot based on a redundancy analysis (RDA); fauna groups of aquatic and soil samples are represented by their 4-letter-abbreviations (see below); arrows refer to the five levels of the environmental variable *‘treatment’*; and site scores are shown with different shapes and colors depending on their treatment affiliation. Axis 1 explains proportionally 11% (*P ≤ 0*.*01*) of the variation in the dataset; Axis 2 accounts for 5% (*n*.*s*.) of the variation. **Abbreviations of animal lineages:** *aquatic fauna*: ACol—Coleoptera Imagos, Anis—Anisoptera Larvae, Anne—Annelida, Brac—Brachycera Larvae, Cera—Ceratopogonidae Larvae, Chir—Chironomidae Larvae, Clad—Cladocera, Cole—Coleoptera Larvae, Cope—Copepoda, Cori—Corixidae, Culi—Culicidae Larvae, divN—Nematocera Larvae (except for Chironomidae and Culicidae), Ephe—Ephemeroptera Larvae, Moll—Mollusca, Nauc—Naucoridae, Nema—Nematoda, Noto—Notonectidae, Ostr—Ostracoda, Plec—Plecoptera Larvae, Zygo—Zygoptera Larvae; *soil mesofauna*: Acar—Acari, Ench—Enchytraeidae, Rest—remaining (not specified) invertebrates from soil samples; *soil nematodes*: Acro—Acrobeles spp., Ceph—Cephalobus spp., Pana—Panagrolaimus spp., Plet—Plectus spp. (all bacterial-feeding), Aphe—Aphelenchoides spp. (hyphal-feeding), Dity—Ditylenchus spp. (plant-associated), Heli—Helicotylenchus spp., Hirs—Hirshamanniella spp., Long—Longidorus spp., Prat—Pratylenchus spp. (all plant-feeding), Dory—Dorylaimus spp., Eudo—Eudorylaimus spp., Prod—Prodorylaimus spp. (all omnivorous), Mono—Monochus spp. (predator).

For the RDA the variable *‘treatment’* (categorical, 5 levels) was included. Based on the total variance in the dataset, the first RDA axis explained 11% (Table D in S1 File Table; *P = 0*.*005*) and represented mostly the ‘straw scattered’ treatment (Fig 5; see also Table E in S1 File—highest absolute value at RDA 1). The second axis accounted for 5% (Table D in S1 File; *P* = *0*.*15*) of the total variance and was related with the ‘straw mixed in’ treatment (Fig 5; see also Table E in S1 File—highest absolute value at RDA 2). In total, 21% of the variance in the dataset was explained by the four constrained RDA axes. Of this variance 52% was explained by RDA 1 and 24% by RDA 2 (Table D in S1 File). The factor *treatment* itself had a significant influence on the abundances of aquatic and soil invertebrates (*P = 0*.*02*; all results of ANOVA permutation tests are given in Table F in S1 File). Finally, we found no significant relationships between litter mass losses and fauna groups as analyses of co-variance and structural equation models did not reveal direct or indirect interaction effects.

## Discussion

### Rice straw decomposition

The present study demonstrates the importance of invertebrate decomposers as ecosystem engineers for sustainable agricultural practices in flooded rice production systems. The rate of decomposition is known to be influenced by a variety of abiotic and biotic factors [3, 20]. According to Singh et al. [18] three main factors are important for efficient residue decomposition in rice-based cropping systems: (1) crop residue factors (like C/N ratios and lignin concentration), (2) edaphic factors (soil properties like moisture content), and (3) management factors. For a long-term sustainable improvement of management practices in paddy fields, it is crucial to understand the ways in which these often interacting factors influence the decomposition of rice straw by invertebrates.

In our experiment, invertebrates contributed to rice straw decomposition as demonstrated by the higher losses of litter biomass from coarse-meshed litterbags compared to fine-meshed ones. This pattern corroborates the results of several previous field studies (e.g. [10, 38]) and supports our first hypothesis that invertebrates contribute to the mass loss of rice straw in paddy fields. The few previous studies dealing with litter decomposition by invertebrates have mostly been conducted in “true” farmers' rice fields, but not under the controlled and comparable conditions as provided at our experimental sites. In general, it may be reasonably assumed that the straw bundled in our litterbags created microsites with a comparably low redox potential that decreased microbial decomposition activity [58]. This implies that our decomposition rates are likely underestimated and that microbial decomposition of dispersed straw is likely higher than that measured in our litterbags. Nevertheless, microbial decomposition of organic matter is less efficient under the anaerobic conditions (e.g. [58]) prevalent in flooded rice fields. In such environments, invertebrate decomposers will ensure sufficient nutrient precipitation from plant residues.

One important step during invertebrate-driven decomposition is straw shredding, which increases the surface area of the rice straw, and therefore the residue-soil contact, and creates a more stable and favorable environment for microbial decomposition [18, 59]. Residue incorporation enhances this effect [18] as is reflected in our results: Litter decomposition with invertebrates (litter mass loss in coarse-meshed litterbags) was significantly faster in fields with rice straw incorporated into the soil compared to fields with rice straw scattered on the field surface. No significant response of solely microbial decomposition (litter mass loss in fine-meshed bags) on management practices was detectable, which indicates that management practices primarily affect decomposition by invertebrates. The process of rice straw decomposition in paddies can be divided into two phases with a rapid phase of decomposition at the beginning (due to leaching and the presence of easily degradable organic C in fresh residues) followed by a slower phase [60]. We found a similar pattern in our experiment (highest litter mass losses after 25 days of incubation), which supports our fourth hypothesis that the abundances of functional groups of invertebrates and their relative contribution to decomposition vary over time with stronger effects at the beginning of the season. It may also explain why differences in litter mass losses between management methods leveled off towards the end of the rice cycle.

It is known that decomposition is related to the C and N contents of plant residues [61]. At the very beginning of the decomposition process, a strong decrease in relative C content due to mineralization processes by microorganisms is common [62]. However, C and N contents in the rice straw litter steadily increased during the time of our experiment which was most likely due to a high leaching of other soluble components, like silicon and potassium [63, 64]. Klotzbücher et al. [46] showed that rice straw from the Laguna region in the Philippines (which we used in our litterbags) has particularly high silicon concentrations, which can amount to nearly five percent of total rice straw dry mass. The reduction of silicon is very fast in paddy fields resulting in higher silicon losses during decomposition compared to C and N mass loss. We found similar changes in C concentration of straw retrieved from both litterbag types which suggests a primarily microbial-driven C-breakdown. In contrast, N contents in the straw differed between fine- and coarse-meshed litterbags and reflected the patterns of the above described litter mass losses: No differences between treatments in straw from fine-meshed bags and generally higher N contents in straw from coarse-meshed bags with lowest values in fields with Ssc treatment. Higher N concentration in the litter may be due to higher fungal biomass [65] which would increase N concentrations creating more attractive conditions for detritivores. This may explain the higher litter mass losses in bags where they had access. This idea is supported by the results of several studies (e.g. [65, 66, 67]) which have reported a positive correlation between decomposition rates and the N contents of plant materials.

Although rice straw is a low-quality resource for decomposers (see Introduction), decomposition rates in rice paddies are rather high. Therefore, N content of the litter is not the only important determinant of litter decomposition rates in wet-rice agriculture. Tian et al. [61] reported that the role of soil fauna is relatively greater in the decomposition of low-quality litter: High C/N ratios as well as high lignin and polyphenol contents decrease the ability of microorganisms to decompose straw. Microbial-driven decomposition is additionally slowed by the anaerobic conditions of flooded rice fields. In fields with ash amendment and with straw mixed into the soil, a lower availability of decomposable plant material in the aquatic phase (where litterbags were set-out) could have led our litterbags to behave as “decomposer baits” leading to a faster colonization of the litterbags by detritivores at the beginning of the experiment. This assumption is supported by the convergence in the loss of rice litter for all five treatments towards the end of the season. In contrast, C and N contents maintained lower levels in the litterbag straw of fields where the rice straw was scattered on the soil surface.

### Invertebrates

Several studies have demonstrated that aquatic invertebrates in rice fields cover the entire spectrum of the freshwater fauna ([36] and references therein). The decomposer fauna in tropical soils consists of morphologically and behaviorally diverse lineages [4] where macro-invertebrates mainly contribute to litter decomposition by burying and shredding of plant material. The positive effects of straw on invertebrate abundances with no effect on the diversity of aquatic or soil invertebrates in the present study is consistent with the findings of Schneider et al. [43] and Hagen et al. [42]. Although the differences between management methods were statistically significant only for the aquatic lineages, the abundances of soil-dwelling lineages showed similar trends in our study. Abundances were consistently highest at plots where straw was scattered on the field surface followed by the fields where the straw was incorporated into the soil. This positive reaction of meso- and macro-invertebrates to straw scattering supports our third hypothesis that the mode of residue application to fields will favor different groups of invertebrates. This is further supported by the findings of Friebe and Henke [44] and Reddy et al. [68], who recorded higher faunal abundances in fields with lower tillage intensity. In contrast, Singh et al. [18] suggested that the incorporation of crop residues into the soil increased populations of all types of macro- and microorganisms in rice fields in India. Whether incorporated or not, the use of rice straw as a fertilizer in irrigated rice cropping systems is beneficial for aquatic and soil invertebrates. We found no positive correlation between the abundance of meso-invertebrates and the decomposition rate. However, such a relationship has been reported by Lekha et al. [1]. Moreover, litter mass losses and the abundances of aquatic fauna showed contrasting patterns in their treatment responses. More long-term experiments will be essential to reveal the relations of invertebrates and their decomposition activity.

Even though the abundance of invertebrates was highest at plots with straw scattered on the field surface, rice plants at these plots produced the lowest yields. Different and partly contradicting short-term effects of practices of crop residue management on rice yields underline the fact that such processes may not manifest within one rice cycle. Xu et al. [69] found no effect of straw amendment on yields in the first season during their experiments, regardless of whether the fields were tilled or not. Long-term experiments by Samra et al. [28], Singh et al. [70] and Thuy et al. [31] also revealed that several crop cycles with continuous residue application are necessary to gain the maximum benefits of straw incorporation.

Despite explaining just a small amount of variance, multivariate analyses indicated that the community composition and abundances of invertebrate lineages differed significantly between our five treatments (straw or ash scattered or incorporated, and control). The graphical illustration of the RDA results revealed a clear separation and clustering of plots with straw treatments. These two straw management methods accounted for the highest amount of variation among all five treatments. Furthermore, their treatment arrows are nearly orthogonal to each other indicating a strong separation of the invertebrate assemblages at these sites. The positioning of the lineages relative to the straw-treatment-arrows shows no ecologically meaningful pattern. However, there are still some obvious trends. Some lineages, like nematodes, cluster primarily in the direction of the arrow representing the straw incorporated management method, while many aquatic lineages seem to favor fields with straw scattered onto the soil surface. Half of these aquatic lineages comprise mainly small plant-, detritus- and bacterial-feeding or omnivorous lineage types (like e.g. *Cladocera* or larvae of *Culicidae* and *Brachycera*); the other half consists of their predators (*Naucoridae*, *Anisoptera* larvae etc.). Thus, straw on the soil surface of rice fields seems to attract small aquatic invertebrates as it provides energetic resources and refuge from predators [71]; this in turn also attracts predatory insects due to an increased prey abundance [42].

Studies like ours will help to unravel the complex mechanisms and interacting effects of faunal abundance, decomposer activity and strategies of crop residue management in tropical flooded rice ecosystems. The next step is the synchronization of plant demand with N fertilization and nutrient release from rice straw residues to reduce the amount of artificial fertilizers that are applied in modern agriculture [20, 31–33], and additionally, to decrease rice straw burning and its subsequent contribution to climate change through air pollutants [72–74].

## Conclusions

Linking farmers’ interests with a sustainable improvement of agricultural practices in compliance with nature conservation is one of the future challenges to stabilize or even increase yields while preserving biodiversity and natural landscape structures. In our study, we demonstrated that invertebrate decomposers contribute substantially to decomposition processes in flooded rice agriculture indicating potential effects on soil fertility and site productivity. Sustainable crop residue management strategies should consider invertebrates when using straw to improve soil conditions. We showed that altering residue management practices prior to cropping significantly influences the litter decomposing activity of invertebrates during the first rice cycle, but we found no effects on microbial-driven decomposition rates. Increasing the rice straw availability in paddy fields, during the aquatic phase as well as in the soil, positively affected the abundances of aquatic and soil fauna groups. Future long-term studies should particularly focus on revealing linkages between litter decomposition by invertebrates and their abundances to evaluate in more detail how crop residue management practices can contribute to the maintenance of ecosystem services provided by invertebrate decomposers in flooded rice ecosystems.

## Supporting Information

S1 Fig C/N ratios: mesh size × time; treatment × time; mesh size × treatment. Comparison of C/N ratios (means + SE) of rice straw litter in (A) coarse- and fine-meshed litterbags at the three retrieval times, (B) bags under the five treatments at the three retrieval times, and (C) coarse- and fine-meshed bags under the five treatments. Different letters above the bars indicate significant differences between means (Tukey’s HSD, *P ≤ 0*.*05*). Asterisks in graph (A) indicate significant differences between the two mesh sizes at one point in time (not between times); *P ≤ 0*.001***. Value of the original straw (= *time 0d*): C/N = 61.5. For abbreviations see Fig 1.
(TIF)

S2 Fig Experimental setup. Experimental setup; treatment abbreviations: ‘Asc’—ash of burned rice straw scattered on the field, ‘Ami’—ash of burned rice straw mixed in the soil, ‘Ssc’—rice straw scattered on the field, ‘Smi’—rice straw mixed in the soil, ‘Ctr’—control (no ash or straw added).
(TIF)

S3 Fig Litter mass loss, N and C content: mesh size × time. Percent litter mass loss (A), N content (B) and C content (C) (means + SE) of rice straw litter in coarse- and fine-meshed bags retrieved at three points in time. Asterisks indicate significant differences between the two mesh sizes at one point in time (not between times); *P ≤ 0*.001***. Values of the original straw (= *time 0d*): N = 0.6%, C = 36.8%.
(TIF)

S4 Fig C & N mass loss: mesh size × time; treatment × time; mesh size × treatment. C-/ N mass loss (g) (means + SE) of rice straw litter in (A/D) coarse- and fine-meshed litterbags at the three retrieval times, (B/E) bags under the five treatments at the three retrieval times, and (C/F) coarse- and fine-meshed bags under the five treatments. Different letters above the bars indicate significant differences between means (Tukey’s HSD, *P ≤ 0*.*05*). Asterisks in graphs (A) and (D) indicate significant differences between the two mesh sizes at one point in time (not between times; *P ≤ 0*.001***). Values of the original straw (= *time 0d*): N = 0.06 g, C = 3.44 g. For abbreviations see Fig 1.
(TIF)

S5 Fig Changes of soil traits and soil fauna abundances. Soil traits: pH (A) and organic C content (B); average numbers of selected soil fauna groups (C-G) per *‘time’*; *P ≤ 0*.05*, *P ≤ 0*.01**, *P ≤ 0*.001***. Abundances of mesofauna (C & D) are given per m^2^ soil area and abundances of soil nematodes (E-G) are given per g soil dry weight.
(TIF)

S1 File Table A in S1 File. The effects of *‘treatment’*, *‘time’*, *‘mesh’* and their interactions on C/N ratios in the rice straw litter using a GLMM type III sum of squares. Significant effects are indicated in bold font. **Table B in S1 File.** The effects of *‘treatment’*, *‘time’*, *‘mesh’* and their interactions on rice straw N and C mass loss using a GLMM type III sum of squares. Significant effects are indicated in bold font. **Table C in S1 File.** The effects of *‘treatment’*, *‘time’* and their interaction on soil traits (pH, organic C content) and selected soil fauna groups using a GLMM type III sum of squares. Significant effects are indicated in bold font. **Table D in S1 File.** Eigenvalues of the four RDA axes and their contribution to the total variance, as well as accumulated constrained (‘Acc.’) eigenvalues and contribution to the accumulated variation of the four RDA axes from the community analyses of aquatic and soil fauna abundances. **Table E in S1 File.** Centroids for factor constraints of the first two RDA axes. Highest absolute values are indicated in bold font. **Table F in S1 File.** ANOVA table of permutation tests for the four RDA axes and the constraining environmental variable *‘treatment’*; ‘Perm’ = number of permutations. Significant effects are indicated in bold font.
(DOCX)

S2 File Raw dataset including all original unchanged data used for the analyses in this study. (XLSX)
