# Supplementary figures and images for: Effects of Residue Management on Decomposition in Irrigated Rice Fields Are Not Related to Changes in the Decomposer Community
Source: PLoS One. 2015 Jul 30;10(7):e0134402. doi: 10.1371/journal.pone.0134402 (PMC4520592; doi:10.1371/journal.pone.0134402)

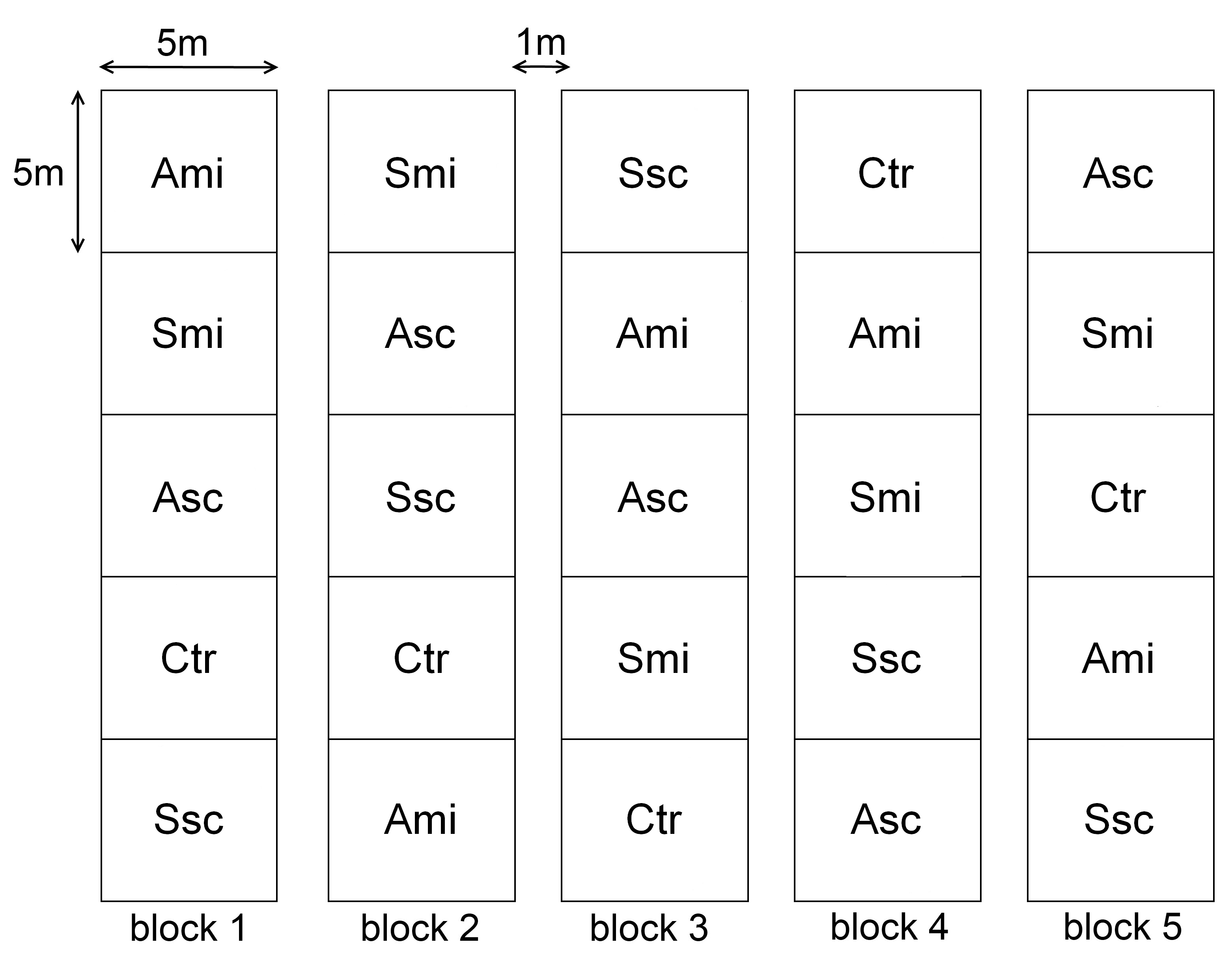

Supplement: S2 Fig — Experimental setup; treatment abbreviations: ‘Asc’—ash of burned rice straw scattered on the field, ‘Ami’—ash of burned rice straw mixed in the soil, ‘Ssc’—rice straw scattered on the field, ‘Smi’—rice straw mixed in the soil, ‘Ctr’—control (no ash or straw added). (TIF) [file pone.0134402.s002.tif]
