# Supplementary material for: Effects of Residue Management on Decomposition in Irrigated Rice Fields Are Not Related to Changes in the Decomposer Community
Source: PLoS One. 2015 Jul 30;10(7):e0134402. doi: 10.1371/journal.pone.0134402 (PMC4520592; doi:10.1371/journal.pone.0134402)
Supplement: S1 File — The effects of ‘treatment’, ‘time’, ‘mesh’ and their interactions on C/N ratios in the rice straw litter using a GLMM type III sum of squares. Significant effects are indicated in bold font. Table B in S1 File. The effects of ‘treatment’, ‘time’, ‘mesh’ and their interactions on rice straw N and C mass loss using a GLMM type III sum of squares. Significant effects are indicated in bold font. Table C in S1 File. The effects of ‘treatment’, ‘time’ and their interaction on soil traits (pH, organic C content) and selected soil fauna groups using a GLMM type III sum of squares. Significant effects are indicated in bold font. Table D in S1 File. Eigenvalues of the four RDA axes and their contribution to the total variance, as well as accumulated constrained (‘Acc.’) eigenvalues and contribution to the accumulated variation of the four RDA axes from the community analyses of aquatic and soil fauna abundances. Table E in S1 File. Centroids for factor constraints of the first two RDA axes. Highest absolute values are indicated in bold font. Table F in S1 File. ANOVA table of permutation tests for the four RDA axes and the constraining environmental variable ‘treatment’; ‘Perm’ = number of permutations. Significant effects are indicated in bold font. (DOCX) [file pone.0134402.s006.docx]

**Table A**

| Factors | C/N ratio | | |
| --- | --- | --- | --- |
|  | Df | F | *P* |
| *treatment* | 4,16 | 0.95 | 0.46 |
| *time* | 2,187 | 346 | **< .0001** |
| *mesh* | 1,206 | 215 | **< .0001** |
| *treatment × time* | 8,187 | 1.14 | 0.34 |
| *treatment × mesh* | 4,206 | 7.40 | **< .0001** |
| *mesh × time* | 2,206 | 20.8 | **< .0001** |
| *treatment × mesh × time* | 8,206 | 1.48 | 0.17 |

Factor *‘treatment*’ represents the five different management practices (Asc, Ami, Ssc, Smi, Ctr; for abbreviations see Fig 1), the factor *‘time’* is the effect of the three different time periods for which the bags were left in the fields (25d, 50d, 75d), and factor *‘mesh’* the two mesh sizes (5 mm and 20 μm) used in every plot. The model also includes the random effects of the factors *‘block’* and *‘replicate’*; these two factors and their interactions are not shown.

**Table B**

| Factors | N mass loss (g) | | |  | C mass loss (g) | | |
| --- | --- | --- | --- | --- | --- | --- | --- |
|  | Df | F | *P* |  | Df | F | *P* |
| *treatment* | 4,16 | 6.42 | **0.002** |  | 4,16 | 4.18 | **0.02** |
| *time* | 2,187 | 836 | **<.0001** |  | 2,187 | 2146 | **<.0001** |
| *mesh* | 1,206 | 176 | **<.0001** |  | 1,206 | 679 | **<.0001** |
| *treatment × time* | 8,187 | 2.70 | **0.01** |  | 8,187 | 5.41 | **<.0001** |
| *treatment × mesh* | 4,206 | 2.61 | **0.04** |  | 4,206 | 9.71 | **<.0001** |
| *mesh × time* | 2,206 | 11.1 | **<.0001** |  | 2,206 | 43.3 | **<.0001** |
| *treatment × mesh × time* | 8,206 | 2.02 | **0.04** |  | 8,206 | 1.31 | 0.24 |

Factor *‘treatment*’ represents the five different management practices (Asc, Ami, Ssc, Smi, Ctr; for abbreviations see Fig 1), the factor *‘time’* is the effect of the three different time periods for which the bags were left in the fields (25d, 50d, 75d), and factor *‘mesh’* the two mesh sizes (5 mm and 20 μm) used in every plot. The model also includes the random effects of the factors *‘block’* and *‘replicate’*; these two factors and their interactions are not shown.

**Table C**

| Factors | Soil pH | | |  | Soil organic C content | | |  | Acari abundance / m^2^ | | |
| --- | --- | --- | --- | --- | --- | --- | --- | --- | --- | --- | --- |
|  | Df | F | *P* |  | Df | F | *P* |  | Df | F | *P* |
| *treatment* | 4,16 | 0.61 | 0.66 |  | 4,16 | 0.49 | 0.74 |  | 4,16 | 1.52 | 0.24 |
| *time* | 1,18 | 4.53 | **0.04** |  | 1,18 | 34.1 | **< .0001** |  | 1,18 | 59.8 | **<.0001** |
| *treatment × time* | 4,18 | 1.10 | 0.39 |  | 4,18 | 0.90 | 0.49 |  | 4,18 | 2.18 | 0.11 |
|  | Soil meso fauna abundance / m^2^ | | |  | Plantfeeding Nematoda abundance / g | | |  | Omnivorous Nematoda abundance / g | | |
|  | Df | F | *P* |  | Df | F | *P* |  | Df | F | *P* |
| *treatment* | 4,16 | 1.52 | 0.24 |  | 4,16 | 1.56 | 0.23 |  | 4,16 | 1.95 | 0.15 |
| *time* | 1,18 | 33.1 | **<.0001** |  | 1,18 | 40.0 | **< .0001** |  | 1,18 | 11.3 | **0.003** |
| *treatment × time* | 4,18 | 3.38 | **0.03** |  | 4,18 | 1.12 | 0.38 |  | 4,18 | 1.45 | 0.26 |
|  | Soil Nematoda abundance / g | | |  |  |  |  |  |  |  |  |
|  | Df | F | *P* |  |  |  |  |  |  |  |  |
| *treatment* | 4,16 | 1.05 | 0.41 |  |  |  |  |  |  |  |  |
| *time* | 1,18 | 7.08 | **0.02** |  |  |  |  |  |  |  |  |
| *treatment × time* | 4,18 | 0.92 | 0.47 |  |  |  |  |  |  |  |  |

Factor *‘treatment’* represents the five different management practices (Asc, Ami, Ssc, Smi, Ctr; for abbreviations see Fig 1) and the factor *‘time’* is the effect of the two sampling dates of soil cores (25d, 75d). The model also includes the random effect of the factors *‘block’*, which is not shown.

**Table D**

|  | RDA 1 | RDA 2 | RDA 3 | RDA 4 |
| --- | --- | --- | --- | --- |
| Eigenvalue | 4.08 | 1.88 | 1.09 | 0.86 |
| Proportion explained (%) | 11.0 | 5.08 | 2.93 | 2.34 |
| Acc. eigenvalue | 4.08 | 1.88 | 1.09 | 0.86 |
| Acc. proportion explained (%) | 51.6 | 23.8 | 13.7 | 10.9 |

**Table E**

|  | RDA 1 | RDA 2 |
| --- | --- | --- |
| Ash mixed in | -0.91 | 0.35 |
| Ash scattered | -0.60 | 0.61 |
| Straw mixed in | 0.51 | **-2.11** |
| Straw scattered | **1.92** | 0.99 |
| Control | -0.92 | 0.16 |

**Table F**

|  | Df | Var | Perm | F | *P* |
| --- | --- | --- | --- | --- | --- |
| RDA 1 | 1 | 4.08 | 199 | 2.81 | **0.005** |
| RDA 2 | 1 | 1.88 | 199 | 1.29 | 0.15 |
| RDA 3 | 1 | 1.09 | 99 | 0.75 | 0.81 |
| RDA 4 | 1 | 0.86 | 99 | 0.59 | 0.93 |
| Treatment | 4 | 7.91 | 199 | 1.36 | **0.02** |
